# Predicting maximum temperatures over India 10-days ahead using machine learning models

**DOI:** 10.1038/s41598-023-44286-1

**Published:** 2023-10-11

**Authors:** J. V. Ratnam, Swadhin K. Behera, Masami Nonaka, Patrick Martineau, Kalpesh R. Patil

**Affiliations:** Application Laboratory, VAIG, Japan Agency for Marine-Earth Science and Technology, 3173-25 Showa-machi, Kanazawa-Ku, Yokohama, Kanagawa 236-0001 Japan

**Keywords:** Atmospheric dynamics, Projection and prediction

## Abstract

In the months of March-June, India experiences high daytime temperatures (Tmax), which sometimes lead to heatwave-like conditions over India. In this study, 10 different machine learning models are evaluated for their ability to predict the daily Tmax anomalies 10 days ahead in the months of March-June. Several model experiments were carried out to identify an optimal model to predict daily Tmax anomalies over India. The results indicate that the AdaBoost regressor with Multi-layer Perceptron as the base estimator is an optimal model to predict the Tmax anomalies over India in the months of March-June. The optimal model predictions are benchmarked against 10-day persistence predictions and the predictions from the Climate Forecast System (CFS) reforecast. The results indicate that the machine learning model skill is higher than persistence and comparable to CFS reforecast 10-day predictions in April and May. In March and June, the machine learning models have low skill scores and perform no better than persistence. These results indicate that the machine learning models are promising tools to predict the surface air maximum temperature anomalies over India in April and May and can complement predictions from more sophisticated numerical models.

## Introduction

**Figure 1:**
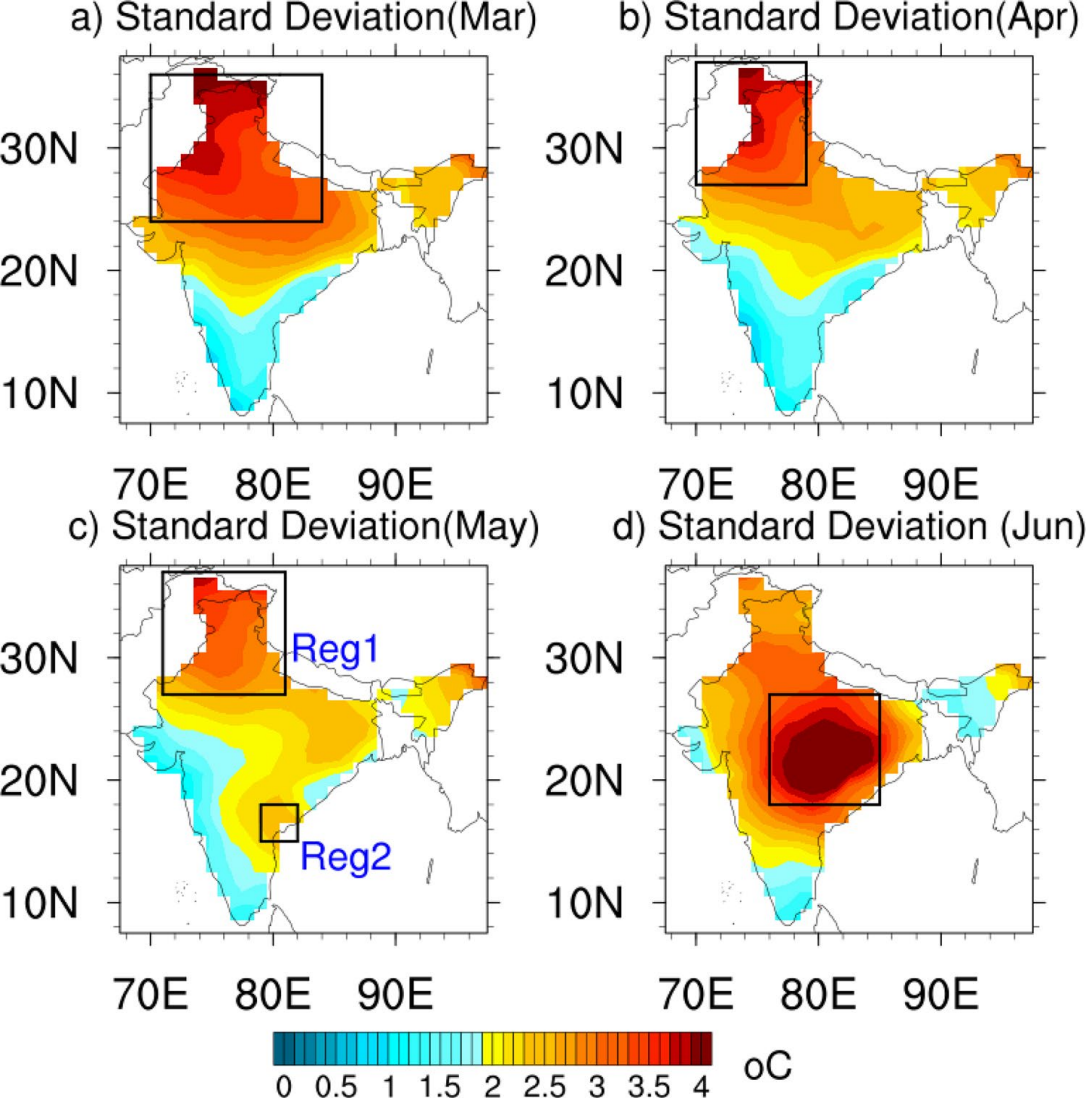
Standard deviation of Tmax over India in (**a**) March, (**b**) April (**c**) May (**d**) June. The regions of large standard deviation (> 2.5 in March–May and > 3.5 in June) are marked with a rectangular box.

India experiences hot weather from March to June with temperatures reaching up to 45 °C on some days. These are also the months in which India experiences heatwaves^1,2^. In recent times there has been an increase in the frequency and persistence of hot days over India and it is projected to increase further in the future^2–4^. Heatwaves cause loss of lives and also affect the economy of the country. Predicting the maximum temperatures at least one week to 10 days ahead would help the planners to prepare well in advance for the eventualities. There have been some efforts to analyze and predict heat waves over India ^5–15^. The studies^5–7^ indicate that the numerical models have reasonable skill in predicting heatwaves over India at least one week − 10 days ahead. The India Meteorological Department (IMD) issues heat wave guidance based on maximum temperature (Tmax) at various time scales based on synoptic analysis of various meteorological parameters and with guidance from several numerical models (https://internal.imd.gov.in/section/nhac/dynamic/FAQ_heat_wave.pdf (page last accessed 1st Aug 2023). In this study, we plan to complement the efforts of the forecasting centers by predicting Tmax using various machine-learning models with a lead time of 10 days. As a first step, we attempt to predict Tmax anomalies only over the regions of large standard deviation (Fig. 1a–d) in Tmax over India in the March-June months as these are regions that are highly prone to heatwaves. The Tmax anomalies over the regions of large standard deviation over India (Fig. 1) are highly correlated with the thermal comfort index, the universal thermal climate index (UTCI ^16^) anomalies with correlation coefficients of 0.74 (Mar). 0.68 (Apr), 0.62 (May Reg1), 0.77 (May Reg2), and 0.91 (June). Also, the Granger causality ^17–21^ test shows the causality relation between Tmax and UTCI to be bi-directional justifying the use of Tmax to predict the heatwaves over the regions of large standard deviation over India (Fig. 1).

In recent times, machine learning and artificial intelligence techniques have been used to model climate and weather at various time scales^22^. The machine learning models to predict extreme events such as heatwaves is still a developing field and only a few studies are available in the literature ^23,24^. However, there has been no systematic evaluation and development of machine learning models to forecast extreme temperatures over India. In this study, we attempt to fill that gap. Before attempting to evaluate machine learning models we carried out experiments with simple linear statistical models to predict the Tmax anomalies at 10-day lead over India but were not successful. So, we attempted to predict the Tmax anomalies using machine learning models, which are non-linear statistical models. We evaluated 10 different machine learning models for their ability to predict Tmax anomalies realistically, by conducting several model experiments by varying the preprocessing techniques, feature reduction using principal component analysis, varying the activation function, and varying the number of neurons used in the models.

## Results

### Skill of the Tmax predictions

The model experiments were evaluated for their skill in predicting the Tmax anomalies over India based on the ACC skill score, RMSE, and their ability to predict extreme Tmax anomalies (exceeding 4 °C). As the skill of the ensemble mean of several models is often higher than that of a single model we generated ensembles of various combinations of model predictions and evaluated their skill in predicting the Tmax anomalies over India in the months March-June. Based on the analysis we identified the configuration of the models with higher ACC and lower RMSE. The model configurations for each month are given in Table 1.

**Table 1:** ACC, RMSE of the predictions and model configuration of the optimum ML model.

	Persistence	CFS	ML model
March	[0.39; 2.97] {0.38; 2.97}	[0.72; 2.00]	[0.27; 2.71] {0.34; 2.63} **Model configuration:** AdaBoost (MLP); Norm; avg of (RELU ADAM, RELU LBFGS, TANH ADAM, TANH LBFGS; nproc:2–20 )
April	[0.15; 3.77] {0.22; 3.45}	[0.49; 2.64]	[0.33; 2.77] {0.35; 2.76} **Model configuration:** AdaBoost (MLP); Norm; PCA; RELU ADAM, avg of nproc:15 and 16
May Reg1	[~ 0.0; 3.53] {0.19; 3.41}	[0.47; 2.3]	[0.33; 2.56] {0.28; 2.80} **Model configuration:** AdaBoost (MLP); Std; PCA; avg of (RELU ADAM, TANH LBFGS; nprocs:10–20)
May Reg2	[0.08; 2.81] {0.18; 2.78}	[0.32; 2.41]	[0.36; 2.42] {0.28; 2.74} **Model configuration:** AdaBoost (MLP); Std; RELU ADAM, avg of nproc: 10–20
June	[0.38; 2.70] {0.32; 2.76}	[0.62; 2.16]	[0.46; 2.25] {0.44; 2.23} **Model configuration:** AdaBoost (MLP); RELU ADAM, avg of (Norm, Norm PCA, Std, Std PCA, Power, Robust; nproc: 2–14)
[corr; rmse] for the period 1999–2020; {corr; rmse} for the period 1982–2020 **corr:** correlation coefficient; **rmse:** root mean square error **Std**: Standardization; **Norm**: Min–Max Normalization **Activation Function:** RELU, TANH; **Solver:** ADAM, LBFGS **nproc:** Number of processors; **PCA:** Principal component analysis **MLP:** Multi layer perceptron			

It is interesting to note that of all the models evaluated in the study the AdaBoost with MLP as the base estimator (hereafter AdaBoost(MLP)) performs better than other ML models (Table 2) in all the months in predicting Tmax anomalies over India (Table 1). Also, as expected we found the ensemble mean of the predictions to be skillful in all the months (Table 1). In March the average of AdaBoost(MLP) predictions with the number of neurons varying from 2 to 20 was found to give optimal results. In April the ensemble average of the predictions from AdaBoost(MLP) input processed using Min–Max normalization and PCA, and configured with RELU activation function and ADAM solver and with the number of neurons varying from 15 to 16 is found to give optimal skill in predicting the Tmax anomalies over India (Table 1). The configurations of the model in the months May and June are given in Table 1.

**Table 2:** List of models and experiments.

Models	(i) Adaptive boosting (Adaboost) and (ii) Bagging regressor (BagReg) with base estimators (a) Decision tree regressor (b) Multi-Layer perceptron (MLP) regressor* (c) Support Vector Machine regressor (SVR) (iii) Gradient Boosting (GBM) regressor (iv) CatBoost regressor (v) Light Gradient Boosted Machine (LightGBM) regressor (vi) XGBoost regressor *Experiments with MLP by varying (a) number of neurons from 2 to 20 for March–May and from 2 to 14 for June (b) activation functions RELU and TANH (c) solvers LBFGS and ADAM
Pre-processing	(i) Standardization, (ii) Min–Max normalization, (iii) Power transformation, (iv) Robust scaler; (v) Principal component analysis (PCA)

In the months of March and June, the persistence predictions have an ACC skill score of about 0.38 (Table 1). In both these months, the CFS reforecast predicted Tmax anomalies have a high ACC skill score of 0.72 and 0.62 whereas the machine learning model does worse than persistence with ACC skill score of 0.27 in the month of March and performs slightly better than persistence in the month of June (Table 1). These findings indicate that the machine learning models used in the study are not much useful in predicting Tmax anomalies over India in the months of March and June.

The ACC skill score of persistent predictions is low in both April and May (Table 1). The machine learning model AdaBoost(MLP) does better than persistence in predicting the Tmax anomalies over India in both of these months and the ACC skill score is lower, but comparable to that of the CFS reforecast 10-day predictions (Table 1). The modest ACC value of the CFS reforecast indicates the 10-day prediction of Tmax anomalies over India in April and May is challenging. The low skill scores of persistence and CFS predictions in April and May maybe indicating a prediction barrier in these months, which needs further investigation. We further evaluated the predictions of machine learning models for the months of April and May and the results are discussed in the following sections.

### Frequency distribution of Tmax predictions

The machine learning model for predicting daily Tmax anomalies should realistically predict both the negative and positive anomalies to be useful for real-time forecasting. So, we compared the first four statistical moments (mean, standard deviation, skewness, and kurtosis) along with the 95% cutoff low and high of the time series of predicted Tmax anomalies with the observed Tmax anomalies of IMD. Models with similar statistical properties to those of IMD Tmax anomalies are considered to be adequate for predictions.

**Figure 2:**
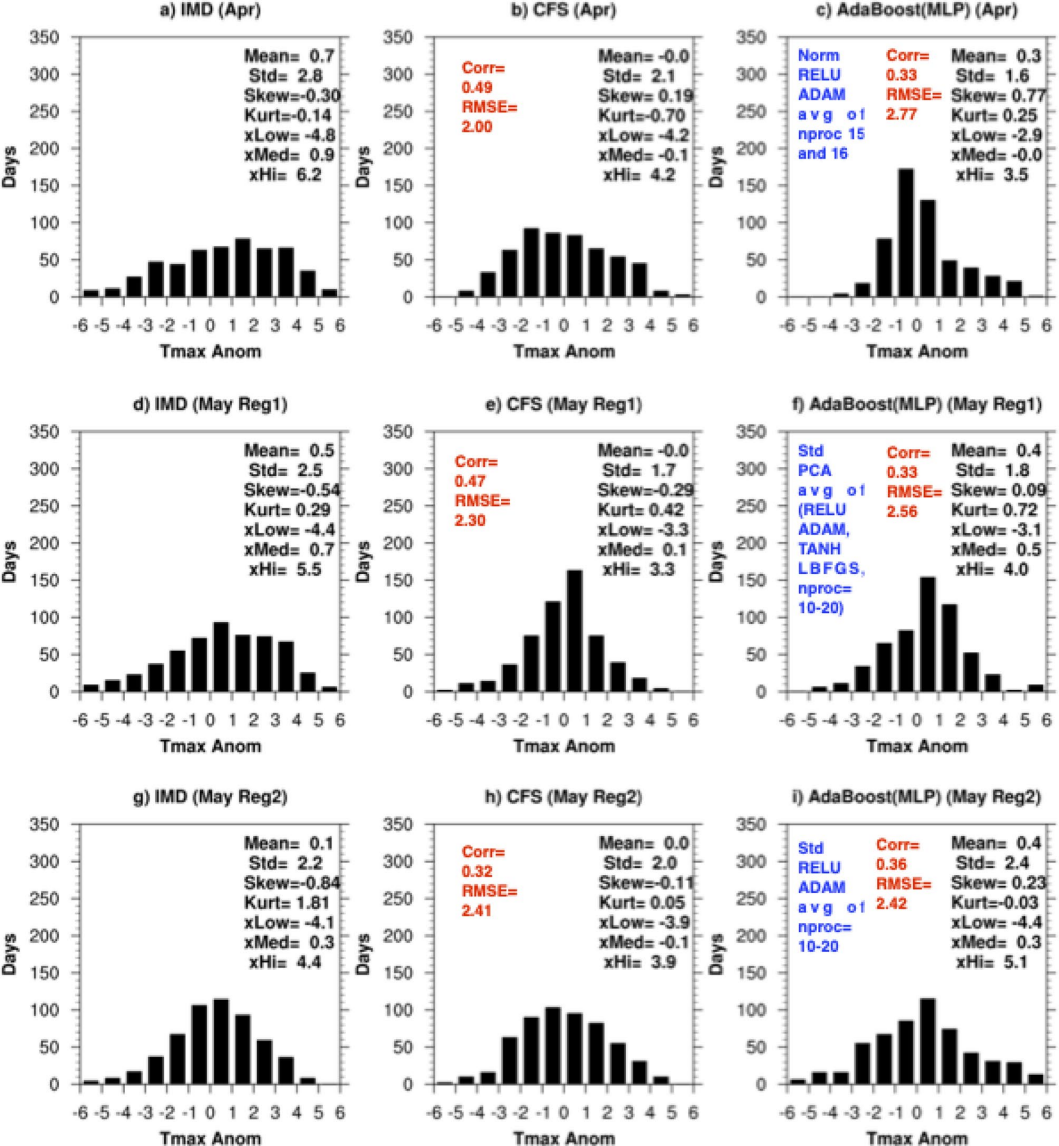
Frequency distribution (Number of days versus Tmax anomaly ranges) of time series of (**a**) IMD, (**b**) CFS, (**c**) AdaBoost(MLP) in April, (**d**) IMD, (**e**) CFS, (**f**) AdaBoost(MLP) in May over Reg1, (**g**) IMD, (**h**) CFS, and (**i**) AdaBoost(MLP) in May over Reg2.

The time series of the IMD area averaged Tmax anomalies over the region of large standard deviation in the northern parts of India (Fig. 1b) in Apr over the period 1999–2020, has a standard deviation of 2.8°C, and is slightly negatively skewed (− 0.30), with kurtosis of − 0.14 (Fig. 2a). The predicted Tmax anomalies in April of both CFS (Fig. 2b) and AdaBoost(MLP) (Fig. 2c) have biases in the first four statistical moments compared to the IMD Tmax anomalies. The Tmax anomalies of the CFS predictions are slightly positively skewed (0.19) with a kurtosis of -0.70 relative to the normal distribution (Fig. 2b). Also, the 95% cutoff low and high values of the predicted time series are small (− 4.2 and 4.2) compared to the IMD values (− 4.8 and 6.2). The AdaBoost (MLP) predicted Tmax anomalies are positively skewed and the model has difficulty in predicting the negative Tmax anomalies (Fig. 2c) in April. The 95% cutoff low and high values (-2.9 and 3.5) are smaller than that of the IMD values.

In May there are two regions of large standard deviation in Tmax, one located over the northern parts of India (Reg1) and the other over the southern parts (Reg2) of India (Fig. 1c). The area-averaged standard deviations of the IMD observed Tmax anomalies over the regions Reg1 and Reg2 are 2.5°C (Fig. 2d) and 2.2°C (Fig. 2g), respectively over the period 1999–2020. The time series over both regions are slightly negatively skewed with values of -0.54 and -0.84. Also, the time series are leptokurtic with Reg2 having a higher value (1.81) compared to Reg1 (0.29) indicating the time series of Reg2 has fatter tails and a narrow peak in the frequency distribution. The 95% cutoff range of Tmax anomaly is also higher over Reg1 (5.5°C) compared to Reg2 (4.4°C) indicating the extreme temperatures over Reg1 to be higher compared to Reg2. The Tmax anomalies of the CFS 10-day predictions over both Reg1 (Fig. 2e) and Reg2 (Fig. 2h) have a 95% high cutoff that is smaller compared to that of IMD value indicating the CFS model fails to predict the extreme temperatures in May. The 95% high cutoff of AdaBoost(MLP) over Reg1 (Fig. 2f) and Reg2 (Fig. 2i) is higher than that of the CFS predicted values though lower than that of the IMD values over Reg11. For Reg2, the AdaBoost(MLP) predicted (Fig. 2i) more days with Tmax anomalies exceeding 5°C compared to the IMD Tmax anomalies (Fig. 2g).

The above analysis indicates the predicted Tmax anomalies have biases in the frequency distribution for various ranges of temperature. However, it is evident from Fig. 2 that the models could generate extreme Tmax anomalies, exceeding 4 °C, in both April and May over India. We analyze those in the following section.

### Hit rate versus False alarm rate

**Figure 3:**
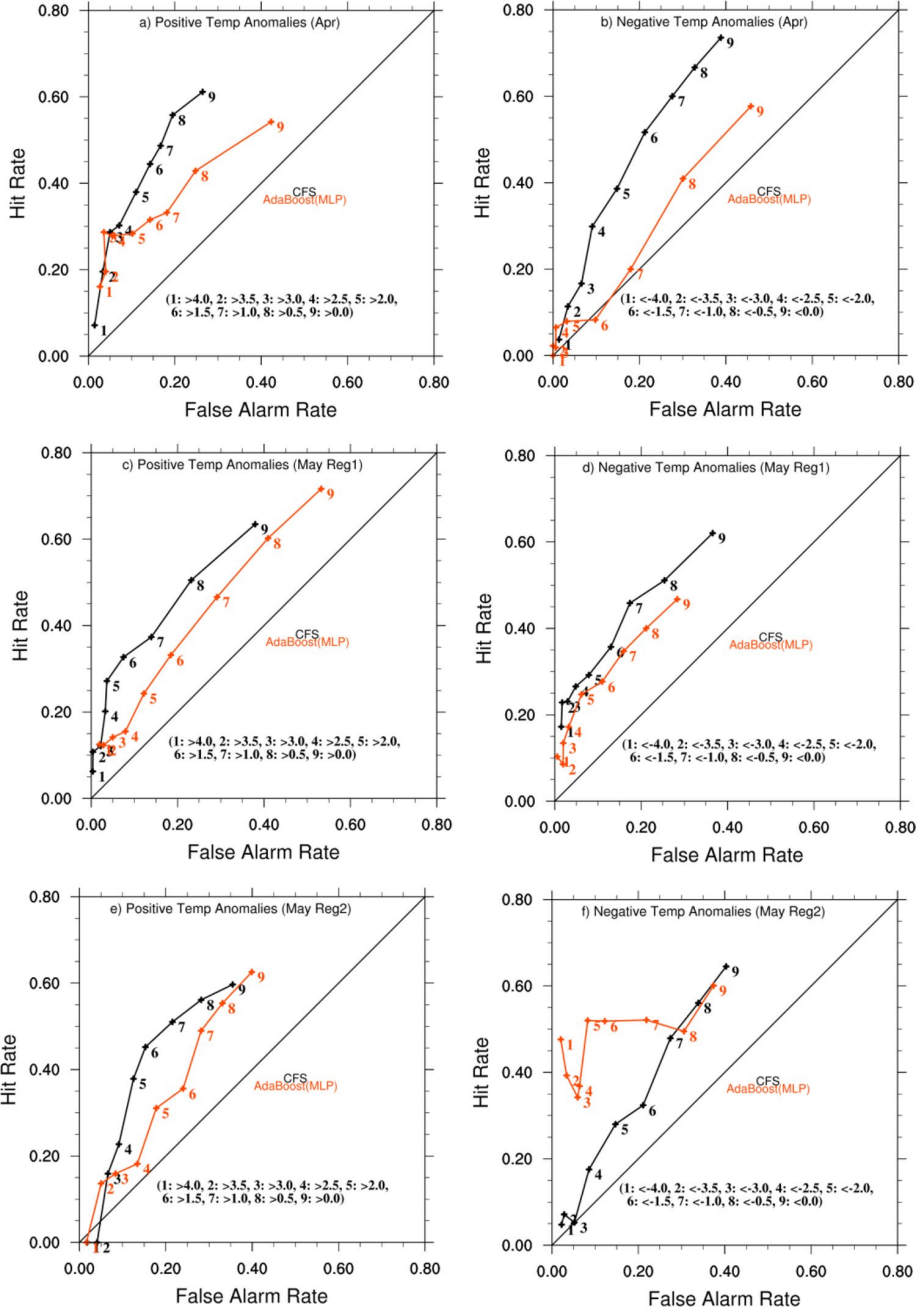
The left and right panels show hit rate vs. false alarm rate for positive and negative temperature anomalies, respectively, for predictions made by CFS (black) and AdaBoost(MLP) (red) models in April (**a**, **b**), May-Reg1 (**c**, **d**), May-Reg2 (**e**, **f**) from top to bottom. The positive temperature anomalies are defined as temperatures above 0.0, 0.5, 1.0, 1.5, 2.0, 2.5, 3.0, 3.5, and 4.0 °C, and are shown with numbers 9, 8, 7….1 on the plot as indicated in each panel. Similarly, the negative temperature anomalies are defined as temperatures below 0.0, − 0.5, − 1.0, − 1.5, − 2.0, − 2.5, − 3.0, − 3.5, and − 4.0 °C, and are also shown with numbers 9, 8, 7….1 on the plot.

The prediction of extreme Tmax anomalies by the models does not guarantee that the model predictions are accurate, as there may be many false alarms in the predicted daily values with mismatches in the predicted daily Tmax anomalies. We examined the hit rate (HR) vs. false alarm rate (FAR)^25^ of the predicted time series to see if the sign and magnitude of the predicted daily Tmax anomalies on a given day matched those of the IMD observed on that day. HR is defined as HR = hit/(hit + miss), where a hit is when an event (Tmax anomaly of particular magnitude and sign on a particular day) occurred and was successfully predicted, miss is when an event occurred but was not predicted, and FAR = (false alarm)/(false alarm + correct rejection), where a false alarm is when an event was predicted but did not occur and correct rejection is when an event did not occur and was not predicted. A prediction is considered to be skillful if HR is greater than FAR. The HR and FAR were calculated for both positive and negative predicted Tmax anomalies for various threshold values. For positive Tmax anomalies the HR and FAR were calculated for the threshold values and the results of the analysis are shown in Fig. 3.

The AdaBoost(MLP) has lower HR compared to CFS for smaller positive temperature thresholds from > 0.0 °C to > 2.5 °C but performs better than the CFS model for thresholds above 3.0 °C (Fig. 3a). For a threshold of > 3.0 °C, the AdaBoost(MLP) model has 35 hits, 87 misses, 18 false alarms and 403 correct rejections, with a HR value of 0.286 and FAR value of 0.03 whereas the CFS model has 35 hits, 87 misses, 21 false alarms and 397 correct rejection with a HR of 0.286 and FAR of 0.05. For threshold > 4.0 °C the AdaBoost(MLP) has HR of 0.16 ( 9 hits and 47 misses) and FAR of 0.02 (13 false alarms and 471 correct rejections) whereas CFS has HR of 0.07 (4 hits and 52 misses) and FAR of 0.01 (7 false alarm and 477 correct rejection) (Fig. 3a). These indicate the AdaBoost(MLP) does slightly better than CFS in predicting the extreme positive Tmax anomalies over India in April. The AdaBoost(MLP) has a large bias in predicting extreme negative Tmax anomalies over India (Fig. 2c) which is also reflected in the plot of HR vs FAR for the negative Tmax anomalies (Fig. 3b). The HR is comparable to FAR for all the ranges of thresholds for the negative Tmax anomalies of the AdaBoost(MLP) model indicating the model has no skill in predicting the negative Tmax anomalies in April. The CFS model has higher HR compared to FAR for all the ranges of negative temperature anomalies (Fig. 3b).

In May, over Reg1, the CFS has higher HR compared to AdaBoost(MLP) for the positive Tmax anomalies for the thresholds from > 0.0 °C to > 3.0 °C (Fig. 3c). However for the thresholds > 3.5 °C and > 4.0 °C the AdaBoost(MLP) has higher HR compared to the CFS reforecast predictions along with higher FAR (Fig. 3c). For threshold > 3.5 °C, AdaBoost(MLP) has HR of 0.12 (8 hits and 57 misses) and FAR of 0.02 (14 false alarm and 479 correct rejection) whereas CFS has HR of 0.11 (7 hits, 58 misses) and FAR of 0.004 (2 false alarm and 491 correct rejection). The AdaBoost(MLP) has an HR of 0.12 (4 hits and 28 misses) and FAR of 0.02 (10 false alarms and 516 correct rejections) and CFS has an HR of 0.06 (2 hits and 30 misses) and FAR of 0.004 (2 false alarm and 524 correct rejection) for threshold > 4 °C (Fig. 3c). The results indicate the performance of CFS and AdaBoost(MLP) is comparable in predicting the extreme positive temperature anomalies though AdaBoost(MLP) has a slightly higher number of false alarms compared to CFS. Both CFS and AdaBoost(MLP) have higher HR compared to FAR over all the thresholds in the predicted negative Tmax anomalies with CFS reforecast performing better with higher HR and lower FAR compared to AdaBoost(MLP) predictions (Fig. 3d).

Both CFS and AdaBoost(MLP) failed to predict the extreme temperatures > 4.0 °C in May over Reg2 (Fig. 3e). The AdaBoost(MLP) has an HR of 0.00 (0 hits and 8 misses) and FAR of 0.01 (9 false alarms and 541 correct rejections) whereas CFS has an HR of 0.00 (0 hits and 8 misses) and FAR of 0.02 (10 false alarms and 540 correct rejection) for temperature threshold > 4.0 °C. For the temperature threshold of > 3.5 the AdaBoost(MLP) has slightly higher HR compared to CFS. The AdaBoost(MLP) has HR of 0.13 (3 hits and 19 misses) and FAR of 0.05 (27 false alarm and 509 correct rejection) whereas CFS has HR of 0.00 (0 hits and 22 misses) and FAR of 0.04 (22 misses and 514 correct rejection) for threshold of > 3.5 °C. For other positive temperature thresholds > 0.0 °C to > 3.0 °C the CFS has higher HR and lower FAR compared to the AdaBoost(MLP) predictions. The AdaBoost(MLP) has higher HR and lower FAR in predicting the negative Tmax anomalies in May over Reg2 (Fig. 3f).

The above analysis shows that the machine learning model AdaBoost(MLP) is suitable for predicting the extreme positive temperatures over India in April and May. AdaBoost(MLP) has performance similar to that of CFS in predicting extreme positive temperatures > 3.5 °C and > 4.0 °C in April and May over India.

### Feature importance of the input attributes in the Tmax anomaly prediction

**Figure 4:**
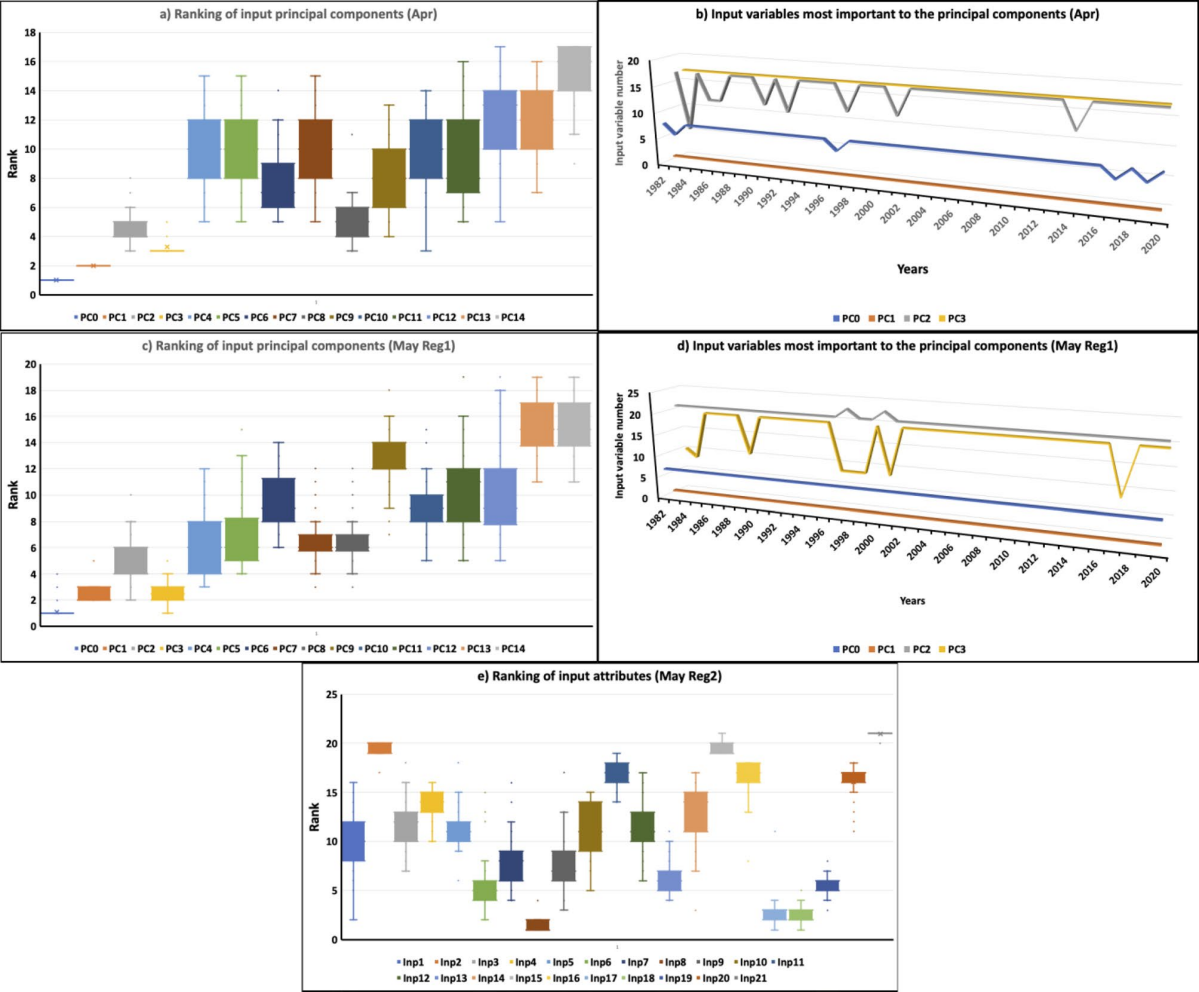
a) Ranking of input attributes using permutation feature importance techniques for April, b) Input variables contributing most to the principal components PC0, PC1, PC2 and PC3 over the period 1982–2020 (**c**) same as (**a**) but for May Reg1 (**d**) same as (**b**) but for May Reg1 and (**e**) same as (**a**) but for May Reg2. The whiskers show the variation of rank in predicting Tmax anomalies from 1982 to 2020.

As discussed in the previous section, the AdaBoost(MLP) model shows good skill in predicting extreme Tmax anomalies over India in April and May. The skills are comparable to the CFS reforecast predictions. In this section we attempt to better understand the features which have contributed to the prediction of the Tmax anomalies in the AdaBoost(MLP) models, thereby getting an idea of the variables important for predicting the Tmax anomalies. For this, we used the permutation feature importance^26^ technique, a tool that is part of scikit-learn software. The permutation importance of an input attribute is calculated by randomly shuffling the attribute and measuring the decrease in model score. A large drop in score indicates the input attribute to be relatively important for the model prediction. After calculating the feature importance of the attributes in predicting Tmax anomalies for all the years 1982–2020, the input attributes were ranked. The feature with the higher rank is considered to have contributed relatively more to the model predictions. The ranking of the input attributes for the identified models and their variation over the years is shown in Fig. 4a–e. As discussed before, the input attributes were derived based on correlation analysis. The correlation does not imply causation. The input attributes may be just statistical artifacts and may not be really responsible for causing variation of Tmax anomalies over India. To identify the input attributes which would have caused the variations of Tmax anomalies over India we applied the Granger causality test to the input attributes with higher ranks (rank < 5) as these input attributes would have a relatively higher effect on the predictions. Granger-causality is a statistical technique that is helpful to determine if one time series is likely to influence the change in another i.e., if one time series can be used to predict the other time series. In our study, we used the “grangertest” function of “lmtest” package of “R software” to implement the Granger causality test. The physical processes through which the input attributes, identified through Granger causality, contribute to the Tmax variations of Tmax over India can be investigated through numerical model experiments, which we intend to carry out in future studies.

In April, PCA was applied to the input after Min–Max normalization before feeding the data to the AdaBoost(MLP) model (Table 1), with preserving 95% of the variance in the data as mentioned in methods section. The number of components selected by the algorithm varied from 15 to 17 for the predictions of April Tmax anomalies for the period 1982 to 2020 using leave-one-year-out cross-validation. The explained variance ratio i.e. the percentage of variance explained by each of the selected components for one of the years is for example 0.2817, 0.1661, 0.0849, 0.0677, 0.0560, 0.0473, 0.0454, 0.0355, 0.0316, 0.0265, 0.0240, 0.0218, 0.0179, 0.0168, 0.0157, 0.0124) ie the PC0 explains about 28.2%, PC1 explains about 16.6%, PC2 explains about 8.5% and so on. We obtained the relative importance of each of the principal components using the permutation feature importance technique and the ranks of the principal components contributing most to the April predictions are shown in Fig. 4a. Only the ranks for PC0-PC14, which are common in all the predictions, are shown in Fig. 4a. As expected, the PC0 which explains a large variance (about 28%) is relatively more important compared to other principal components, followed by PC1, PC3 and PC2. The PC4-PC15 contributions have large variations in the ranking in the predictions (Fig. 4a). After identifying the relatively important principal components we identified the input variables which are most important to those principal components by using the “component_” attribute of Scikit-learn PCA implementation. The input variable with a large “explained_variance_” is output by the attribute and considered the most important input variable contributing to that principal component. We obtained such values for all the predictions and the results are shown in Fig. 4b.

**Figure 5:**
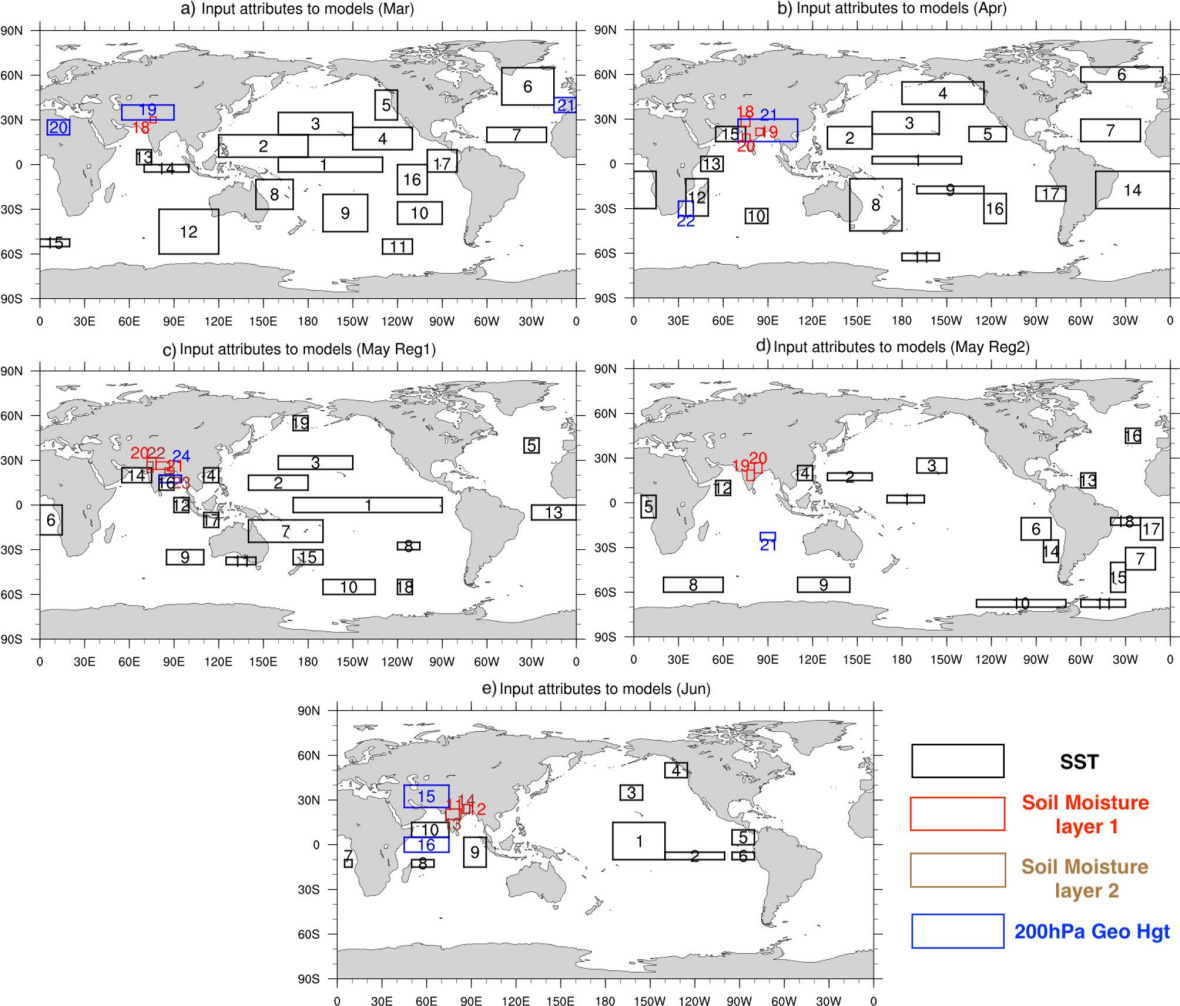
Regions of significant correlation between the Tmax anomalies over the regions of large standard deviation over India (marked in Fig. 1) and spatial distribution of 10-day lead SST, Soil moisture, and 200 hPa geopotential height anomalies in (**a**) March, (**b**) April, (**c**) May (Reg1), (**d**) May (Reg2) and e) June. The area averages of the variables over the regions indicated by the boxes are the input attributes to the models.

The input attribute over region 6 and region 8 of Fig. 5b contribute most to the principal component PC0 (Fig. 4b), region 1 contributes most to PC1, region 12 and region 17 contribute most to PC2 though region 6 and 11 contribute in two of the years (Fig. 4b) and region 17 contributes most to PC3 (Fig. 4b). We verified using the Granger causality test if the input from these identified regions can Granger cause Tmax anomalies over India. Of the identified regions we find that regions 6, 8, and 17 can Granger cause Tmax anomalies over India. For other regions (1 and 12) the causality test is not statistically significant so it is difficult to explain physically how the input from these regions would have contributed to the prediction of Tmax anomalies over India. The physical mechanism through which SST over regions 8 and 17 can cause variation in Tmax anomalies over northern parts of India is not clear and needs model experiments to clarify their influence. The SST variation over region 6 is mostly a response to the variation in the atmospheric processes and those atmospheric processes can propagate to the northern parts of India and cause variations in the temperature over India^27^.

PCA was applied to the input after standardization before providing the data to the AdaBoost(MLP) model (Table 1) in May for predicting Tmax over Reg1. The number of components selected by the algorithm varied from 15 to 17 for the predictions of May Tmax anomalies over Reg1 for the period 1982 to 2020 using leave-one-year-out cross-validation.

The relative importance of each of the principal components using the permutation feature importance technique and the ranks of the principal components contributing most to the May predictions are shown in Fig. 4c. Only the ranks for PC0-PC14, which are common in all the predictions, are shown in Fig. 4c. The PC0 which explains a large variance (about 25%) is relatively more important compared to other principal components, followed by PC1, PC3, and PC2.

The regions 7, 1, 21, 23,19, 8, and 10 shown in Fig. 5c contribute most to PC0, PC1, PC2, and PC3 (Fig. 4d). Granger causality test shows regions 1, 7, 19 and 10 can Granger cause Tmax anomalies over India. Region 1, located over the equatorial Pacific (Fig. 5c), can affect the Tmax anomalies over India through an atmospheric teleconnection in response to the heating associated with the SST anomalies over region 1 and can extend to the northern parts of India and affect the Tmax variations over Reg1 in May. However, the physical processes through which the other regions can cause variations of Tmax over India need to be understood through numerical model experiments.

Of the regions shown in Fig. 5d, SST anomalies over regions 8, 18, 17, 6 19, and 13 are relatively more important in the prediction of Tmax anomalies over India in May over Reg 2 compared to the input from other regions (Fig. 4e). The Granger causality test showed that regions 6 of the above six regions to Granger cause Tmax anomalies over the coastal regions of India. The physical processes through which the SST anomalies over region 6 can affect the Tmax anomalies over India are not clear and careful model experiments are needed to understand the physical processes.

## Discussion

In this study, we attempted to predict daily Tmax anomalies over the regions of large standard deviation in Tmax over India using machine learning models in the months of March-June. We validated 10 machine learning models to check their usefulness in predicting the Tmax anomalies. We carried out several model experiments varying the preprocessing method of input data time series, experiments with feature reduction using PCA, varying the activation function, and varying the number of neurons in the models with MLP as the base estimator.

Based on the analysis of statistical moments of the predicted time series of the Tmax anomalies, on the correlation between the predicted Tmax anomalies and IMD observed Tmax anomalies, and RMSE between the predicted and observed Tmax anomaly time series, we tried to identify an optimal model. As we had a large number of predictions from model experiments, we generated ensembles by using various models and evaluated them to identify an optimal model. The results showed the ensemble average of AdaBoost with MLP as the base estimator with varying numbers of neurons to outperform the other 9 machine learning models and their ensemble averages with a higher hit rate and a lower false alarm rate at extreme temperatures in the months of March–May. The correlation coefficient of the predicted time series by the optimal model is modest (Table 1) but is statistically significant at the 99.9% confidence level using Student’s 2-tailed test due to a large number of data points in each month, March, May Reg1, May Reg2 has 1209 data points and Apr, June has 1170 data points. Benchmarking the AdaBoost(MLP) results with the persistence and CFS reforecast 10-day predictions showed the AdaBoost(MLP) to perform better than persistence in predicting Tmax anomalies in April and May over India. The performance of AdaBoost(MLP) is also similar to that of CFS in predicting extreme temperatures in April and May. However, AdaBoost(MLP) does no better than persistence in March and June indicating the model to be not much useful in predicting Tmax anomalies over India in those months. The results indicate the machine learning models can complement the existing state-of-the-art numerical models in predicting the Tmax over India in the months of April and May, the months in which the numerical models also have difficulty in generating useful predictions.

The analysis based on the permutation feature importance showed the regions of relative importance in the prediction of Tmax anomalies over India. Using the Granger causality test showed some of these relatively important regions to Granger cause Tmax anomalies over India. In April, the SST over the North Atlantic is found to be relatively important in predicting the Tmax anomalies over India. In May (Reg1) the SST related to ENSO is found to be one of the relatively important inputs to predict Tmax anomalies. The physical processes through which other relatively important input attributes contribute to the variation of Tmax anomalies over India are not clearly understood. It would be interesting to carry out numerical model experiments to understand them.

One of the caveats of this study is that we predict only the area averaged value of the Tmax anomalies over the regions of large standard deviation in Tmax over India. In the future, we plan to extend this work to predict Tmax anomalies over all the grid points covering India.

The Tmax anomaly predictions in this study are solely based on input attributes derived from observed or analyzed estimates of SST, soil moisture, and 200 hPa geopotential height anomalies. A vast amount of data from the numerical weather prediction models are available which can be used to train the machine learning models to improve the prediction of Tmax anomalies. Also, using a more complex deep learning model trained on observed and numerical weather prediction model output may help in improving the prediction of Tmax anomalies with higher skill. We plan to carry out such hybrid model studies in the future.

## Methods

### Models

In this study, ten different machine learning models (Table 2) are evaluated for their ability to predict 10-day lead daily Tmax anomalies over India in the months March-June. The study covers the period 1982–2020, the period of availability of the IMD Tmax data. The machine learning models validated in this study are the AdaBoost (Adaptive Boosting)^28,29^ regressor with (i) Decision tree regressor^30^ (ii) Multi-layer Perceptron (MLP)^31^ regressor and (iii) Support Vector Machine regressor (SVR^32^) as the base estimators; (iv) Gradient Boosting regressor (GBM)^33^; (v) CatBoost regressor^34^ (vi) Light Gradient Boosted Machine (LightGBM)^35^ regressor (vii) XGBoost^36^ regressor; and Bagging regressor^37^ (BagReg hereafter) with (viii) Decision tree regressor (ix) MLP (x) SVR as base estimators. The GBM, CatBoost, LightGBM, and XGBoost use a tree-based regressor as the base estimator. All the above models are implemented using the Scikit-learn^38^ toolbox (https://scikit-learn.org/stable/). A detailed description and implementation details of the machine learning models can be found on the scikit-learn webpages and in the cited references.

The MLP is one of the widely used machine learning techniques in climate science^39–46^. The MLP regressor consists of an input layer, a hidden layer, and an output layer. The model predictors are fed to the input layer, non-linear relations between the input predictors are obtained in the hidden layer, and the weights obtained from the hidden layer are used to predict and output results through the output layer. The results of the MLP regressor are sensitive to the choice of the number of neurons used in the hidden layer. The results of MLP regressor are also sensitive to the choice of the activation function and the solver. In this study, we carried out several experiments by varying the number of neurons, the activation function, and solvers.

The SVR is also extensively used for prediction in climate sciences^47–49^. The SVR obtains a non-linear relation between the input predictors and the weights of the relation are used to predict the future values. In this study, we used the SVR with ‘rbf’ kernel and the kernel coefficient ‘gamma’ with the default ‘scale’ option. The regularization parameter ‘C’ is set to the default value of 1.

The decision tree regressor predicts the target variable from the regression tree developed from the predictors based on certain decisions. The tree is developed from the predictors with the least mean square error. We used the default values of the tunable parameters specified in the Scikit-learn toolbox for the decision tree regressor.

To improve the skill and robustness of the MLP, SVR, and decision tree regressor predictions, ensemble methods such as averaging and boosting are used. In the averaging method, several predictions are generated by sampling the predictors and the average of the predictions is generated. The ensemble-averaged predictions are better than any single prediction because it reduces variance and hence overfitting. The “Bagging regressor” belongs to the class of averaging methods. The boosting methods combine several weak learners to produce a powerful ensemble i.e., first a model is built from the training data, then a second model is built to reduce the errors in the first model. This process is continued sequentially till model bias is reduced. The “AdaBoost”, “GradBoost”, “CatBoost”, “LGBM”, and “XGBoost” belong to the class of boosting methods. In this study, AdaBoost and Bagging regressor with MLP, SVR, and decision tree as base estimators are evaluated. “GradBoost”, “CatBoost”, “LGBM” and “XGBoost” have a decision tree as the base estimator. The number of estimators for Bagging and Boosting was set to 100 in our study. All the boosting algorithms were used with default values for the tunable parameters.

Several model experiments were carried out to identify a machine learning model predicting daily Tmax anomalies realistically. The preprocessing of the input attributes to bring them to a similar scale is an essential step in machine learning. There are several techniques to preprocess the data, such as standardization, min–max normalization, power transformation, and robust scaler technique. In the standardization technique, the mean of each input attribute is removed and then each attribute is scaled by the standard deviation of the attribute. The mean and standard deviation of the training data set are first calculated and then the whole dataset (training + testing data) is standardized with the obtained standard deviation. In min–max normalization, the whole data set is brought within the range [0,1]. In the Robust scaler method, the median is first removed and the data is scaled according to the quantile range, which makes this method robust to outliers. In the power transform method, a power transformation is applied to each input attribute to make the data more gaussian-like, which stabilizes the variance, and minimizes skewness. In all the preprocessing techniques, scaling parameters are obtained from the training data, and applied to both the training and test datasets. All four preprocessing techniques were applied to scale the input attributes in all the months of Mar-Jun and input to all the 10 models thereby generating many experimental model predictions for evaluation.

Feature reduction techniques such as principal component analysis (PCA)^50^ are often found to be useful in improving the skills of machine learning models. PCA is a statistical technique to convert high-dimensional data to low-dimensional data by retaining the data which explains most of the variance. The PCA was applied to the input predictors to reduce the features. We used the PCA technique to generate experimental predictions for all 10 models. Model experiments were generated by preprocessing the input attributes by standardization and min–max normalization before applying the PCA to reduce the features and then input to the models. The features which explain 95% percent of the variability are used as input to the models (n_components = 0.95). By setting “n_components = 0.95” in the Scikit-learn PCA implementation, the algorithm chooses the number of components that explain 95% of variance.

The MLP has an option to increase the number of neurons. We varied the number of neurons from 2 to 20 to generate additional model experimental predictions for March–May and from 2 to 14 for the June predictions. The range for varying the number of neurons was decided based on the number of input attributes to the MLP model (Fig. 5). The MLP results are also sensitive to the activation function and the solver. We tested two activation functions viz. TANH and RELU^51^ and two solvers ‘LBFGS’ (Limited-memory Broyden-Flecher-Goldfarb-Shanno algorithm) and ‘ADAM’ ^52^ with the models with MLP as base estimators. In summary, the model experiments for each month were configured by (i) varying the preprocessing method, (ii) feature reduction using PCA, and (iii) varying the number of neurons, activation function and solver in the MLP. The first two (i, ii) were applied to all 10 models and (iii) was applied to those models with MLP as the base estimator. In total, 960 model predictions were generated for each of Mar-May months and 670 for June. The evaluated model experiments in the study are tabulated in Table 2 for clarity. We evaluated these experimental predictions to identify a model with reasonable skill in predicting Tmax anomalies in those months.

Leave-one-year-out cross-validation is used to generate the predictions. For example, to predict the Tmax anomalies of March 2020, we use the daily data of March from 1982 to 2019 for training and predict the daily Tmax anomalies of all 31 days of March 2020 with the trained models. This process is repeated to obtain the predictions of March for all the years from 1982 to 2020. A similar technique is applied to obtain the daily predictions for the months of April, May, and June.

### Predictors

**Figure 6:**
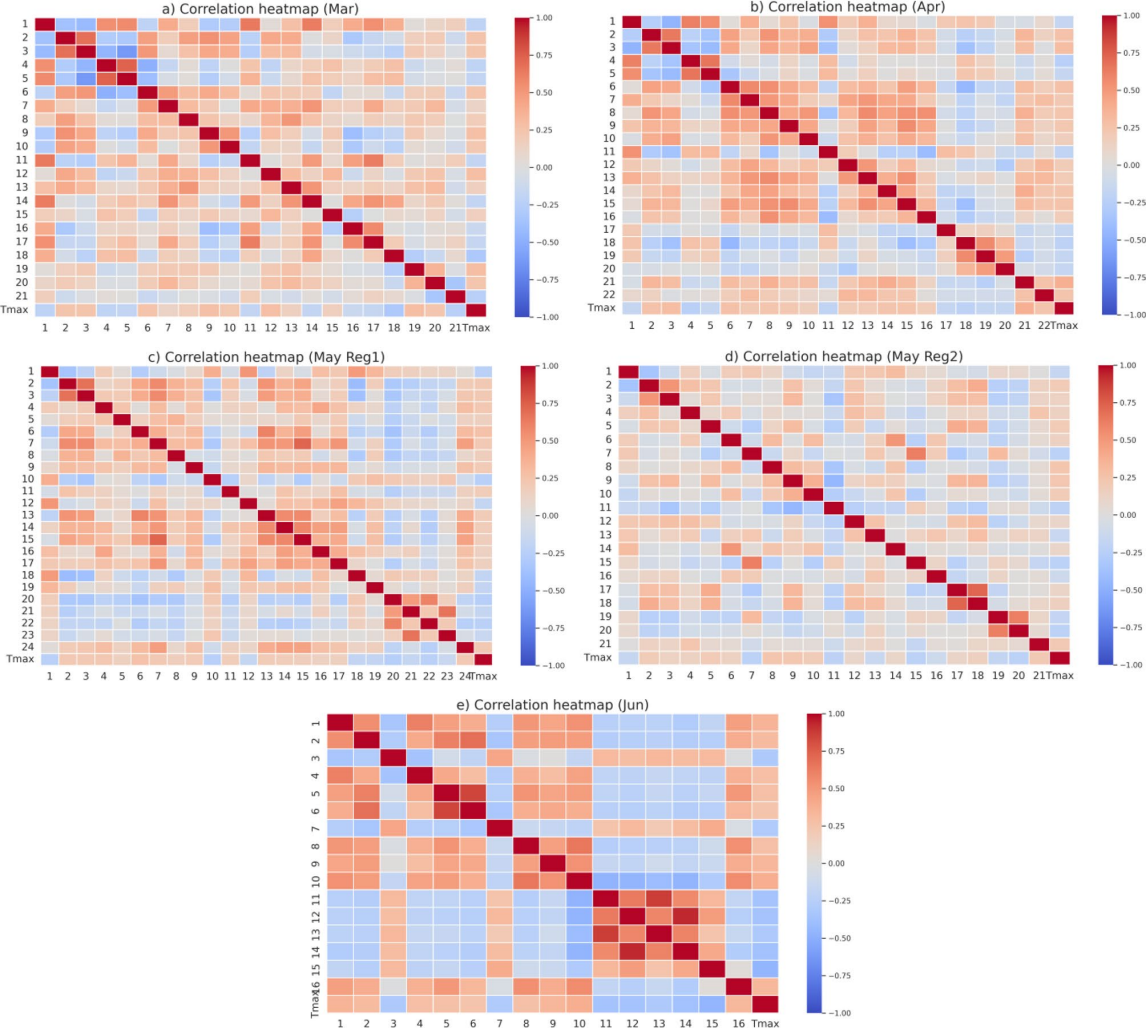
(**a**–**e**) Correlation heatmap showing the correlation between the input attributes in March, April, May (Reg1), May(Reg2) and June.

The hot weather conditions over India can be partly explained by variations in the sea surface temperature (SST) in the equatorial Pacific ^2^ and variations in the blocking events over high latitudes^27^. The variations in the SST in the equatorial Pacific affect the precipitation over India and thus the quantity of soil moisture over the Indian landmass, though with a lag of several months. Therefore, we use these variables at a lead time of 10 days as the input attributes or predictors for the machine learning models to predict Tmax anomalies. The input attributes are derived from the SST, soil moisture, and 200 hPa geopotential height anomalies based on the correlation between the regions of large standard deviation in Tmax in each month (rectangular region marked in Fig. 1) and 10-day lead SST, soil moisture and 200 hPa geopotential height anomalies. The correlated regions, with statistical significance at 99.9% level using Student’s 2-tailed t-test, in the months March-June are shown in Fig. 5. The area average of the statistically significant regions, shown in Fig. 5, is given as the input to the machine learning models. There are 21 input attributes in March (Fig. 5a), 22 in Apr (Fig. 5b), 24 (21) input attributes in May for prediction over Reg 1 (Reg2) (Fig. 5c, d), and 16 input attributes for prediction of Jun (Fig. 5e) Tmax anomalies. A correlation heatmap of the input attributes (Fig. 6a–e) shows the input attributes to have low correlation coefficients with each other in all the months Mar–June indicating the identified input attributes to be independent and useful as predictors for the machine learning models. The Tmax dataset from IMD^53^ was used in this study for training and validation of the machine learning models. The daily Tmax data is at a horizontal resolution of 1° × 1° and covers the period of the study 1982 to 2020. The daily NOAA OI SST V2 High-resolution dataset^54^ along with the daily ERA5 global reanalysis^55^ variables (soil moisture and geopotential height at 200 hPa) were used in the study. The daily SST and ERA5 datasets were interpolated to 1° × 1° horizontal resolution to match the resolution of Tmax dataset. The daily SST and soil moisture were smoothed using a 5-day running mean as the daily observed/reanalyzed values of these slowly varying variables are noisy. The daily anomalies of all the variables Tmax, SST, soil moisture, and 200 hPa geopotential were derived by removing their respective daily climatology (base period 1982–2020).

### Climate forecast system reforecast

The results of the predictions from the machine learning models are benchmarked against 10-day (i) persistence forecasts and (ii) the Climate forecast system (CFS) reforecasts. Persistence forecasts are obtained by assuming that the Tmax anomalies observed on the forecast initial date persist for the next 10 days. The models with smaller anomaly correlation coefficient (ACC) and higher root mean square error (RMSE) than the persistence forecast are considered to have no skill.

The U.S. National Centers for Environmental Prediction (NCEP) CFS reforecasts were produced with the operational CFS version 2 (CFSv2) model^56^ at a resolution of T126. The CFS reforecasts are initialized with CFS reanalysis at 00z, 06z, 12z, and 18z and the predictions are generated for the next few months for each initial condition. We used the 10-day predictions of Tmax from these reforecasts in this study. The 10-day predictions from the CFS reforecast data are available from 1999 to 2020 for all the months March-June. However, the 10-day predictions in the years 2005–2008 are missing on several days when last accessed (data was last accessed on the NCEI site https://www.ncei.noaa.gov/products/weather-climate-models/climate-forecast-system on 19th May 2023). We compared the predictions of the machine learning models with the CFS 10-day predictions for the years 1999–2020 discarding the predictions for the missing years 2005–2008.

## Data Availability

Data used for analysis and machine learning model input are openly available. The Tmax data from IMD, India is available from https://www.imdpune.gov.in/lrfindex.php (on the page one has to click the “Gridded data Archive” button to go the download page) (page last accessed 1st August 2023). The SST data is available from https://psl.noaa.gov/data/gridded/data.noaa.oisst.v2.highres.html (page last accessed 1st Aug 2023). The ERA5 soil moisture, 200hPa geopotential height and UTCI data are available from Copernicus Climate Change Service (C3S) climate data store (https://cds.climate.copernicus.eu/#!/search?text=ERA5&type=dataset; page last accessed 1st August 2023). The CFSreforecast 10-day predictions are available from the webpage https://www.ncei.noaa.gov/products/weather-climate-models/climate-forecast-system (last accessed 1st August 2023).
